# Exploring Fear of Cancer Recurrence in a Sample of Heterogeneous Distressed Cancer Patients with and Without a Psychiatric Disorder

**DOI:** 10.1007/s10880-021-09776-2

**Published:** 2021-06-17

**Authors:** Else M. Bisseling, Félix R. Compen, Melanie P. J. Schellekens, Belinda Thewes, Anne E. M. Speckens, Marije L. van der Lee

**Affiliations:** 1 Department of Psychiatry, Radboud University Medical Centre, 6525 EX Nijmegen, The Netherlands; 2 Department of Psychiatry, Radboud University Medical Centre for Mindfulness, Reinier Postlaan, Postbus 9101, 6500 HB Nijmegen, The Netherlands; 3 Centre for Psycho-Oncology, Scientific Research Department, Helen Dowling Institute, 3723 MB Bilthoven, The Netherlands; 4 School of Psychology, University of Sydney, Sydney, NSW 2006 Australia; 5 Department of Medical and Clinical Psychology, School of Social and Behavioral Sciences, Tilburg University, Warandelaan 2, 5037 AB Tilburg, The Netherlands

**Keywords:** Cancer, Oncology, Psychiatric disorder, Psychological distress, Fear of cancer recurrence

## Abstract

Fear of Cancer Recurrence (FCR) is a concern among cancer patients. Recent insights suggest that FCR should be viewed as a distinct syndrome. However, few studies have explored its overlap with psychiatric morbidity. We examined this overlap in a sample of distressed cancer patients. Self-referred patients (*n* = 245) were assessed with the Structured Clinical Interview for DSM-IV-TR Axis-I disorders and the Fear of Cancer Recurrence Inventory-Short Form. Proportions of patients with and without a psychiatric disorder meeting validated cut-offs for screening and clinically relevant FCR were compared. The prevalence of psychiatric disorders was 36%. Clinically relevant FCR was found in 198 patients (81%). Patients with a current psychiatric disorder reported clinically relevant FCR more frequently (89%) compared to those with no disorder (77%). Of patients reporting clinically relevant FCR, the majority (61%) did not additionally meet the criteria for a psychiatric disorder. These findings suggest that there should be particular attention for patients with elevated levels of FCR, warranting FCR-specific treatment.

*Trial registry number* Clinicaltrials.gov NCT02138513

## Introduction

Fear of cancer recurrence (FCR) is defined as the “Fear, worry, or concern relating to the possibility that cancer will come back or progress” (Lebel et al., 2016). FCR is one of the most prevalent areas of unmet needs in cancer patients (Simard et al., 2013). It has been positively associated with other self-report measures of anxiety symptoms, intrusive thoughts and avoidance and negatively associated with quality of life in a large sample of cancer patients (Simard et al., 2013). A systematic review on FCR in adult cancer patients (Simard et al., 2013) showed that across different cancer sites, 39–97% of cancer patients reported some degree of FCR, 22–87% reported a moderate to high degree, and 0–15% reported severe levels of FCR.

There is debate in the literature whether or not FCR is part of psychiatric disorders and if it should be seen as a distinct syndrome warranting specific intervention. In some countries, like the Netherlands, this debate is becoming significant since evidence-based psychosocial interventions are only reimbursed by health insurers for cancer patients with diagnosable psychiatric disorder.

Thewes and colleagues demonstrated in a sample of 218 young women with breast cancer (Thewes et al., 2013), that most women with screening levels of FCR did not additionally meet screening criteria for generalized anxiety disorder or hypochondriasis. Therefore, the authors suggested that FCR should be viewed as a distinct syndrome warranting specific treatment.

However, the relationship between Fear of Cancer Recurrence and established psychiatric disorders is not well understood. Concerns about FCR are among the first symptoms reported by cancer patients who developed a major depression (Kleiboer et al., 2011). Worry and rumination are considered to be the core cognitive processes in anxiety and depressive disorders (Fresco et al., 2002; Nolen-Hoeksema, 2000), which are more prevalent in cancer patients than in the general population (Hartung et al., 2017; Mitchell et al., 2011).Therefore, worry and fear associated with cancer recurrence might be part of known psychiatric disorders such as a major depressive disorder and anxiety disorder.

While several studies have examined the overlap between FCR and psychological symptoms in specific cancer types, only self-report symptom scales or screening instruments were used for assessing psychiatric disorders (Mirabeau-Beale et al., 2009; Roth et al., 2006; Thewes et al., 2013). Only three studies, with varying results, have examined the relationship between FCR and psychiatric disorders using gold standard psychiatric interview methods. These studies report that 6% to 40% of patients with elevated FCR have comorbid psychiatric disorders (Dinkel et al., 2014; Simard & Savard, 2015; Skaali et al., 2009). However, their findings should be interpreted with caution, as the included studies either measured FCR with one item, FCR was rated with one question measure asking: “During the last week, have you been afraid of relapse of your disease?” (Skaali et al., 2009), used a small sample size (*n* = 60) (Simard & Savard, 2015) or only anxiety disorders and not depressive disorders were assessed (Dinkel et al., 2014). Furthermore, patients with metastases were excluded from all the above studies. Therefore, generalizability is limited.

The aim of this study is to contribute to understanding of the relationship between Fear of Cancer Recurrence and established psychiatric disorders by (1) comparing the mean level of FCR in cancer patients with and without a psychiatric disorder; and (2) comparing the proportion of patients with and without psychiatric morbidity who meet validated criteria for screening or clinically relevant FCR.

## Methods

### Patients and Procedure

Participants were recruited as part of a three-armed multicenter randomized controlled trial comparing group-based Mindfulness-Based Cognitive Therapy (MBCT) and individual internet-based MBCT to Treatment As Usual (TAU) (Compen et al., 2018). Inclusion criteria were: (a) a cancer diagnosis, any tumor or stage; (b) ≥ 11 on the Hospital Anxiety and Depression Scale (HADS (Bjelland et al., 2002; Spinhoven et al., 1997); (c) computer literacy and internet access; (d) a good command of the Dutch language. Exclusion criteria were: (a) severe psychiatric condition as acute suicidal ideation or psychosis; (b) change in psychotropic medication dosage within a period of three months prior to baseline; and (c) current or previous participation in a mindfulness-based intervention (**≥**4 sessions of MBCT). The study was approved by the local ethics committee and registered on May 6th 2014 (Clinicaltrials.gov NCT02138513). Participants were recruited in participating specialized mental health care institutes for psycho-oncology, via social media, patient associations and advertorials in local newspapers in The Netherlands. Participants who were interested in participation could enroll themselves at the study website at which point they completed the screening assessment (HADS). Participants with HADS ≥ 11 were contacted by telephone by one of the researchers to assess eligibility. Written informed consent was obtained prior to participation. A subsequent research interview was conducted in which the Structured Clinical Interview for DSM-IV-TR Axis-I disorders (SCID-I; First et al., 2012) was administered to diagnose possible psychiatric disorders. Participants completed remaining (self-report) questionnaires online prior to randomization.

### Assessments

#### Demographic and Clinical Information

The following demographic and clinical characteristics were assessed: gender, age, marital status, education, cancer diagnosis, time since cancer diagnosis, current anticancer treatment, current psychopharmacological treatment.

#### Psychiatric Disorder

Presence of DSM-IV Axis-I psychiatric disorder was assessed by the *Structured Clinical Interview for DSM-IV-TR Axis-I disorders* (SCID-I; First et al., 2012). The screening module and sections on current and past (recurrent) depressive disorder, current anxiety disorder, and current adjustment disorder were used. The SCID-I was administered by trained interviewers with a master in Behavioral Science (FC) and two Masters-level psychology students. All interviews were audio-taped. Two experienced psychiatrists (EB and AS) and one experienced psychologist (ML) supervised the administration of all the SCID-I interviews and double-rated (*n* = 98) all the audiotapes with positive answers on the 13 item screening module. When there was a discrepancy, the rating was discussed together until consensus was reached.

#### Fear of Cancer Recurrence

Severity of FCR was assessed with the *Fear of Cancer Recurrence Inventory-Short Form* (FCRI-SF*;* (Simard & Savard, 2009a)). The FCRI is a multi-dimensional measure intended for use with cancer patients in all stages and currently one of the strongest measures available. The 9-item severity subscale (total range 1–36), also named FCRI-SF, and has been recommended as a screening tool for high FCR. A cut-off score of ≥ 13 on the FCRI-SF is associated with the highest sensitivity rates for discovering the presence of significantly high levels of FCR, which enables it to efficiently screen for FCR. A cut-off score of ≥ 16 on the FCRI-SF is validated as the optimal cut-off point to identify the presence of severe levels of FCR to distinguishing clinically relevant FCR (Simard & Savard, 2015). In the current study, the Dutch translation of the FCRI was used (FCRI-SF-NL;(Van Helmondt et al., 2017)). Internal consistency in the present sample was high, Cronbach’s alpha = 0.90.

#### Differences in Symptoms Measured with FCRI and SCID-I.

The FCRI was developed by a committee of experts in psycho-oncology and based on a cognitive-behavioral conceptualization of FCR. This instrument assesses how severe the fear of recurrence is, what triggers it, how patients cope with it, how much distress they experience from it, whether it results in functioning impairment, whether patients have insight that they worry too much and how they seek reassurance.

When assessing an adjustment disorder the onset of emotional or behavioral symptoms must occur in response to an identifiable stressor, and within 3 months of the stressor. Distress must be disproportionate to the severity or intensity of the stressor, taking into account contextual and cultural factors and it has to lead to significant impairments in social, occupational or other domains of functioning. The difference with FCR is that FCR can occur outside this timeframe and although the distress related to it may be often understandable and therefore not easily judged to be disproportionate, it may at the same time be so severe that it interferes with functioning. The main difference with depressive disorder is the duration criterion that depressed mood must last at least two weeks and be there most of the day, nearly every day. And the criteria for depressive disorder include a range of somatic symptoms like weight loss, sleep problems, which are not assessed within the FCRI. The main difference with generalized anxiety disorder is again the duration criterion (at least 6 months), and criteria such as being easily fatigued or have difficulty concentrating. The criterion difficulty controlling the worry has overlap with FCR, but in FCR the content of the worry is recurrence, whereas in generalized anxiety disorder, the content of worry is much broader. Thus, the criteria according to DMS are more specific in duration and type of symptoms than the items in the FCRI.

### Data Analysis

Statistical analyses were run in IBM SPSS Statistics version 22. Descriptive statistics were used for the sociodemographic and clinical characteristics, and prevalence rates of current psychiatric disorders. One way ANOVA’s with post hoc test were performed to compare mean levels of FCR between groups. A one-way ANOVA was performed with FCR severity as the dependent variable and psychiatric disorder (no versus yes) as fixed effect. A second ANOVA was performed with FCR severity as the dependent variable and type of psychiatric disorder (no/depression disorder/anxiety disorder/adjustment disorder) as fixed effect. Post Hoc Bonferonni tests were conducted to assess whether level of FCR severity differs between the different types of psychiatric disorders. The homogeneity of variance and normality assumption were met.

Proportions of participants with and without a psychiatric disorder meeting validated cut-off for screening and clinically relevant FCR were compared using logistic regression. If a sociodemographic or clinical variable significantly predicted the level of FCR, this variable is added as a control variable in the ANCOVA or logistic regression.

## Results

Of 245 cancer patients eligible for the RCT the majority was female (85.7%), diagnosed with breast cancer (61.6%) and treated with a curative intent (84.1%). Mean age of the sample was 51.7 years (SD = 10.7), and mean time since diagnosis was 3.5 years (SD = 4.7). Almost half of participants (47.3%) had current anticancer treatment; of those, one-third (32.2%) had long-term hormonal treatment. See Table 1 for demographic and clinical characteristics.

**Table 1:** Demographic and clinical characteristics of the total sample and divided in absence/presence of psychiatric disorder

	Total (*n* = 245)	No psychiatric disorder (*n* = 156)	Psychiatric disorder (*n* = 89)	
	*M (SD)*	*M (SD)*	*M (SD)*	*P*
Age, *M* (*SD*)	51.7 (10.7)	51.8 (10.8)	51.4(10.5)	.797
Time since diagnosis (years)	3.5 (4.7)	3.4 (4.0)	3.6 (5.7)	.755
Psychological distress level				<.001
HADS total	17.7 (6.6)	15.2 (5.8)	21.9 (5.7)	
Fear of cancer recurrence				.011
FCRI-SF	21.3 (6.5)	20.5 (6.7)	22.7 (5.9)	
	*N (*%)	*N(*%)	*N (%)*	
Gender				.665
Male	35 (14.3)	21(13.5)	14 (15.6)	
Female	210 (85.7)	134(86.5)	76 (84.4)	
Relationship				.143
Yes: Married, living together	202 (82.4)	132 (85.2)	70 (77.8)	
No: Divorced / widow/ single	43(17.6)	23 (14.8)	20 (22.2)	
Educational level				.575
Low/Intermediate	79 (32.2)	48 (31.0)	31(34.4)	
High	166 (67.8)	107 (69.0)	59 (65.6)	
Diagnosis				.179
Breast	151(61.6)	97 (62.2)	54 (60.0)	
Gynecological	18 (7.3)	14 (9.0)	4 (4.4)	
Colon	12 (4.9)	9 (5.8)	4 (3.3)	
Non-Hodgkin	11(4.5)	8 (5.2)	3 (3.3)	
Prostate	16 (6.5)	10 (6.5)	6 (6.7)	
Other	37 (15.1)	17 (11.0)	20 (22.2)	
Treatment intent				.399
Curative	206 (84.1)	128 (82.6)	78 (86.7)	
Palliative	39 (15.9)	27 (17.4)	12 (13.3)	
Active anticancer treatment				.962
None	129 (52.7)	81 (52.3)	48 (53.3)	
Hormone	79 (32.2)	48 (31.0)	31 (34.4)	
Chemotherapy/Radiation	12(4.9)	8 (4.1)	4 (4.4)	
Other	23 (10.4)	18 (1.6)	7 (7.7)	
Psychopharmacological treatment				<.001
Yes	70 (28.6)	31(19.9)	39 (43.8)	
No	175 (71.4)	125 (80.1)	50 (56.2)	

There were no differences in socio-demographics between participants with or without any psychiatric disorder (see Table 1). However, concerning clinical characteristics, participants with Axis-I psychiatric disorders reported significant higher levels of psychological distress (M = 21.9, SD = 5.7) compared to those without a psychiatric disorder (M = 15.2, SD = 5.8 (SE = 0.76, *p* = 0.001). In addition, they used psychopharmacological medication more often (43.8%) compared to those without a psychiatric disorder (19.9%) χ2 (1, *n* = 245) = 15.9, *p* < 0.001. The mean score on FCRI-SF in the entire sample was 21.3 (SD = 6.5).

None of the sociodemographic and clinical characteristics significantly predicted FCR levels, except for treatment intent. Participants in palliative care reported significantly higher severity levels (*p* = 0.002) on the FCRI-SF (M = 24.2, SD = 6.4), than those who were treated with curative intent (M = 20.7, SD = 6.4).

### Psychiatric Morbidity

Of the SCID-I interviews with the 245 participants, 89 (36%) participants met the criteria for a current psychiatric disorder. Of those, 42 (47%) met the criteria of a depressive disorder, 27 (30%) had an anxiety disorder, 20 (23%) had an adjustment disorder. Five (6%) participants had concurrent depressive and anxiety disorders (see Table 2).

**Table 2:** Prevalence of psychiatric disorders and mean level of FCR

	N	% (95%CI)	M (SD) FCR
No disorder	156		20.5
Any disorder	89	100	22.7 (5.9)
-Depressive	42	47.2 (37–57)	22.3(6.1)
-Anxiety	27	30.3 (22–41)	23.3 (4.8)
-Adjustment	20	22.5 (15–32)	22.8 (6.8)

### Mean Levels of FCR in Cancer Patients with and Without Psychiatric Diagnosis

On average, participants with a psychiatric disorder reported significantly higher severity levels (*p* = 0.011) on the FCRI-SF (M = 22.7, SD = 0.62), than those without a current psychiatric disorder (M = 20.5, SD = 6.7) (see Table 3). Post hoc Bonferonni tests revealed that mean levels of FCR did not significantly differ between specific groups of psychiatric disorders (no disorder, depressive disorder, anxiety disorder, adjustment disorder), F = 2.318, *p* = 0.076, η2 = 0.025. Adding treatment intent (curative versus palliative) as a control variable in the ANCOVA’s resulted in similar findings.

**Table 3:** Fixed effects ANOVA results with FCRI-SF as dependent variable

Predictor	Sum of squares	Df	Mean square	F	*P*	*η*^*2*^
Intercept	104,786,03	1	104,786,03	2531.77	.000	
Psychiatric Disorder	274,16	1	274,16	6.62	.011	.025
Error	10,015,99	242	41,39			

### Screening Level (High) FCR and Clinically Relevant (Severe) FCR in Cancer Patients with and Without Psychiatric Diagnosis

Two hundred and twenty-one participants (91%) scored above the FCRI-SF screening cut-off (≥13) (see Table 4). Almost all participants (97%) with a current Axis-I disorder scored in the high range (≥13) for FCR. Thus participants with any Axis-I disorder were significantly more likely to score above FCRI-SF screening cut-off (≥13) than participants without an Axis-I disorder, (OR = 4.17, *p* = 0.024). Adding treatment intent (curative versus palliative) as a control variable in the logistic regression analysis resulted in similar findings.

**Table 4:** Screening level (high) FCR and clinically relevant (severe) FCR in cancer patients with and without psychiatric diagnosis

	No disorder	Disorder			
	(*n* = 156) *n* (%)	Any Disorder (*n* = 88)^a^ *n* (%)	Depressive (*n* = 42) *n* (%)	Anxiety (*n* = 26)^a^ *n* (%)	Adjustment (*n* = 20) *n* (%)
Screening level					
% < 13 FCR (*n* = 23)	20 (12.8)	3 (3.4)	2 (4.8)	0 (0.0)	1 (5.0)
% ≥ 13 FCR (*n* = 221)^b^	136 (87.2)	85 (96.6)	40 (95.2)	26 (100.0)	19 (95.0)
Clinically relevant					
% < 16 FCR (*n* = 46)	36 (23.1)	10 (11.4)	5 (11.9)	2 (7.7)	3 (15.0)
% ≥ 16 FCR (*n* = 198)^b^	120 (76.9)	78 (88.6)	37 (88.1)	24 (92.3)	17 (85.0)

^a^*n* = 26 rather than 27 (see Table2) because one person with an anxiety disorder did not fill out the FCRI. ^b^This cumulates to 244 because one participant did not fill out the FCRI

Regarding this level of high FCR, the majority of participants (61%) did not additionally meet the criteria for a psychiatric disorder. As almost all of the participants scored above the screening cut-off, the proportion of clinically relevant (severe) FCR was also examined in this sample. Clinically relevant (severe) FCR (≥16) was found in 199 participants (81%). Most participants (89%) with a current Axis-I disorder scored in the clinically relevant range (≥16) for FCR. Participants with any Axis-I disorder were significantly more likely to score above the threshold for clinically relevant FCR (≥16) than participants without an Axis-I disorder, OR = 2.34, *p* = 0.028. Adding treatment intent (curative versus palliative) as a control variable in the ANCOVA’s resulted in similar findings. Regarding this level of severe FCR, the majority of participants (62%) did not additionally meet the criteria for a psychiatric disorder.

## Discussion

The aim of our study was to better understand the relationship between FCR and psychiatric morbidity by describing the prevalence and nature of psychiatric morbidity, comparing the mean level of FCR in heterogeneous cancer patients with and without psychiatric diagnosis and exploring the overlap of high and severe FCR with psychiatric morbidity.

We found significant differences in mean levels of FCR between participants, such that having a psychiatric disorder or being in a palliative stage was associated with elevated mean levels of FCR. Similar to previous studies in curative treated cancer patients, the prevalence of psychiatric disorders in this sample was approximately 40% (Kuhnt et al., 2016; Mitchell et al., 2011). Cancer patients in palliative care were found to be at greater risk for developing elevated levels of FCR, but a palliative stage of disease was not associated with increased likelihood of psychiatric disorder. Over 80% of the sample showed clinically relevant FCR. This is higher than has been reported in a review on FCR in general cancer populations (49%) (Simard et al., 2013) and higher than previously found in a population of breast cancer patients (70%) (Thewes et al., 2012) and could be explained by the fact that this sample consisted of help-seeking distressed cancer patients.

Our findings confirm earlier research findings that FCR is a common distressing phenomenon in cancer patients (Fardell et al., 2016, 2017; Simard et al., 2013), and that it is highly prevalent in patients with psychiatric morbidity (Mitchell et al., 2011; Simard & Savard, 2009b, 2015; Thewes et al., 2013) and patients in palliative care (Wal et al., 2016). While the vast majority of patients with psychiatric disorder experience elevated levels of FCR, the majority of patients with elevated FCR do not experience psychiatric disorder. We replicated Simard’s findings (Simard & Savard, 2015) that high FCR is comorbid with psychiatric morbidity over one-third of cases. It was found that high FCR is comorbid with depressive disorders as well as anxiety disorders. It could be that having high levels of FCR might be a prodromal or atypical symptom of a range of Axis-I psychiatric disorders in cancer patients. Therefore, longitudinal studies are needed to disentangle the causal and temporal relationship between FCR and psychiatric morbidity.

As clinically relevant FCR was found in 80% of cases in the present sample, a higher cut-off, even up to ≥22 (Fardell et al., 2017), might be needed to clearly distinguish cancer patients in need of more intensive FCR-specific interventions.

### Study Strengths and Limitations

A major strength of this study was the use of the gold standard to assess psychiatric disorders. Two experienced psychiatrists and one experienced psychologist supervised and double-rated the SCID-I interviews and particular emphasis was placed on detecting psychiatric disorders. Another strength was that this research was conducted in a relative large sample of heterogeneous cancer patients with respect to cancer type and prognosis. Despite these strengths there are some noteworthy limitations. The present study was conducted in a group of distressed help-seeking cancer patients. Although this patient-centered nature of recruitment resulting in a convenience sample might benefit generalization of research findings to clinical practice, the self-selection inherent in this sampling method threatens internal validity and can be seen as a drawback, limiting the extent to which the present findings generalize to the broader population of cancer patients. We did not assess past anxiety disorders while it could be that high FCR is another manifestation of for example Generalized Anxiety Disorder, but with the anxiety focused on cancer recurrence. Future research should assess past anxiety disorders to know whether high FCR is a clinical manifestation of pre-existing anxiety disorder.

### Clinical Implications

Fear and anxiety are part of cancer survivorship. Specific worry about cancer recurrence is one of the most common concerns of cancer patients. We found that the majority of cancer patients with high FCR do not have psychiatric morbidity, suggesting that FCR is a distinct syndrome, worthy of specific interventions. It is to be discussed whether high FCR warrants a separate DSM category, or that it fits in within existing categories like illness anxiety. We recommend that future studies will also look at the criterion of significant impairments in social, occupational, or other domains of functioning specifically. Patients with elevated levels of FCR might benefit from a stepped care approach, involving strategies such as watchful waiting or psycho-education for low to moderate levels of FCR, self-management interventions for moderate to high levels of FCR, and evidence-based face-to-face psychological interventions (Butow et al., 2017; Herschbach et al., 2010; Smith et al., 2015) or blended therapy approaches (van de Wal, et al., 2017) for patients with severe FCR. Therefore, greater accessibility of FCR-specific treatment programs is a priority. In some countries, evidence-based psychosocial interventions are only reimbursed by health insurers for cancer patients with diagnosable psychiatric disorder. The findings of the present study have specific relevance for the way in which FCR interventions are funded and how they are implemented. The present study found that over one-third of cases of severe FCR overlaps with Axis-I psychiatric morbidity. Therefore, whenever necessary, referral to a clinical psychologist or psychiatrist should be offered when severe FCR levels are identified.
